# Role of endoplasmic reticulum stress in apoptosis of differentiated mouse podocytes induced by high glucose

**DOI:** 10.3892/ijmm.2014.1642

**Published:** 2014-02-03

**Authors:** YANPING CAO, YONGMEI HAO, HANG LI, QINGJUAN LIU, FENG GAO, WEI LIU, HUIJUN DUAN

**Affiliations:** 1 Department of Pathology, Hebei Medical University, Shijiazhuang, Hebei 050017, P.R. China; 2 Department of Nephrology, The First Hospital of Handan, Handan, Hebei 056002, P.R. China; 3 Department of Endocrinology, The Second Hospital of Hebei Medical University, Shijiazhuang, Hebei 050000, P.R. China; 4 Department of Histology and Embryology, Hebei Medical University, Shijiazhuang, Hebei 050017, P.R. China; 5 Department of Pathology, The Third Hospital, Hebei Medical University, Shijiazhuang, Hebei 050051, P.R. China

**Keywords:** endoplasmic reticulum stress, unfolded protein response, podocytes, apoptosis, diabetic nephropathy

## Abstract

Podocytes are terminally differentiated epithelial cells lacking the ability to proliferate. The loss of podocytes is a hallmark of progressive kidney diseases, including diabetic nephropathy (DN). Endoplasmic reticulum stress (ERS)-induced apoptosis is involved in a number of pathological conditions, including DN. The aim of the present study was to investigate whether a high glucose environment induces the apoptosis of podocytes through ERS. Differentiated mouse podocytes were divided into three groups: the normal glucose group (NG, 1 g/l D-glucose), the high glucose group (HG, 4.5 g/l D-glucose) and the mannitol group (M, 1 g/l D-glucose plus 24.4 mM mannitol). The cells were harvested following stimulation with the indicated treatments for 12, 24, 48 and 72 h. Podocyte apoptosis was determined using TUNEL assay and flow cytometry (propidium iodide staining). Glucose-regulated protein 78 (GRP78), CCAAT/enhancer-binding protein (C/EBP) homologous protein (CHOP/GADD153) and caspase-12 expression was analyzed by RT-PCR, western blot analysis and immunocytochemistry. The apoptotic rate increased significantly in the HG group compared with the NG and M groups at 48 and 72 h (all P<0.01). GRP78 expression, an indicator of ERS, was increased from 12 h, indicating that ERS was activated. Subsequently, two ER-associated death (ERAD) pathways, the CHOP/GADD153- and caspase-12-dependent pathways, were detected. CHOP/GADD153 expression reached its peak at 48 h, and caspase-12 expression gradually increased with time. Spearman’s correlation analysis revealed that caspase-12 and CHOP/GADD153 positively correlated with the apoptotic rate (r=0.915, P<0.01 and r=0.639, P<0.01). Our results demonstrated that hyperglycemia (high glucose) induced apoptosis partly through ERS in the differentiated mouse podocytes, which possibly contributes to the pathogenesis of DN.

## Introduction

Diabetic nephropathy (DN) is one of the most common microvascular chronic complications associated with diabetes, and is the most common cause of end-stage renal disease (1,2). DN develops over several years, and involves characteristic pathological changes, such as the excessive accumulation of extracellular matrix (ECM), glomerulosclerosis, tubule dilatation and atrophy, as well as interstitial fibrosis (3). Previous studies have focused on the accumulation of the ECM (4). A growing body of evidence suggests that glomerular podocytes are key players in DN (5). Podocytes are terminally differentiated cells unable to regenerate and the loss of podocytes results in permanent alterations in the glomerular filtration barrier (6,7).

The endoplasmic reticulum (ER) plays a critical role in controlling the fate of cells. The ER is a highly dynamic organelle responsible for multiple cellular functions. However, the ER is highly sensitive to alterations in its homeostasis. A number of conditions can disturb ER functions, and these conditions induce a state known as ER stress (ERS) (8,9).

The hyperglycemia-induced increase in reactive oxygen species (ROS) has been shown to be involved in podocyte apoptosis and depletion *in vitro* and *in vivo*, suggesting that podocyte apoptosis/depletion represents a novel early mechanism leading to DN (10,11). There are direct correlations between ERS and oxidative stress (12). Accumulating evidence indicates that ERS-related apoptosis may be involved in β-cell loss in type 1 and 2 diabetes mellitus (13,14).

A number of signaling pathways have evolved to cope with ERS. The first response involved is the unfolded protein response (UPR), in which ER chaperone proteins are upregulated, which may alleviate ERS (15). Glucose-regulated protein 78 (GRP78) is the main modulator of UPR. It can bind to ER sensors, such as protein kinase R (PKR)-like ER kinase, inositol requiring 1 (IRE1) and activating transcription factor 6 (ATF6), inhibiting their activation (16). GRP78 plays a critical role in the recognition of unfolded proteins (16).

If these adaptive responses cannot alleviate ERS, apoptosis is triggered by various pathways that are not yet fully understood. However, two pathways of ER-associated death (ERAD) have been defined, characterized by the activation of CCAAT/enhancer-binding protein (C/EBP) homologous protein (CHOP/GADD153) and of caspase-12 (8,17).

However, the molecular mechanisms underlying the development of DN remain to be clarified. We hypothesized that ERS is involved in high glucose (HG)-induced podocyte apoptosis. The aim of this study was to examine the expression of GRP78 and the ERAD pathways (CHOP/GADD153- and caspase-12-dependent pathways) in podocytes exposed to a HG environment. The results revealed that podocytes treated with HG underwent ERS, which presumably is an adaptive, protective UPR reaction for cell survival; however, this protective effect was short-lived, since the continued exposure to HG eventually overpowered this protective effect, leading to apoptosis. These results indicate that novel (previously unkown) mechanisms involved in DN may be targeted by novel therapeutic interventions.

## Materials and methods

### Podocyte culture

Conditionally immortalized mouse podocytes purchased from the Cell Culture Center (Peking Union Medical College, Beijing, China) were cultured as previously described (6). In this cell line, a temperature-sensitive SV40 large T-cell antigen (tsA58 Tag) is controlled by a γ-interferon inducible H-2Kb promoter. To induce proliferation, the cells were grown on type I collagen-coated plastic culture bottles (BD Biosciences, Bedford, MA, USA), at 33°C in RPMI-1640 culture medium (Gibco-BRL, Gaithersburg, MD, USA) supplemented with 10% fetal bovine serum (FBS; Gibco-BRL), 100 U/ml penicillin and 100 mg/ml streptomycin (both from Invitrogen, Carlsbad, CA, USA), to which recombinant mouse γ-interferon 10 U/ml (PeproTech, Rocky Hill, NJ, USA) was added (growth-permissive conditions). To induce quiescence and phenotype differentiation, the podocytes were grown at 37°C and deprived of γ-interferon (growth-restrictive conditions) in RPMI-1640 supplemented with 10% FBS, and 1–2 drops of penicillin and streptomycin. The culture medium was replaced every three days. When the cells grew slowly, the cell volume increased significantly, and pedicels (foot processes) extended from the podocytes, visualized under a phase contrast microscope (Olympus, Tokyo, Japan), indicating that the podocytes had differentiated. When the podocytes reached 75–85% confluence under growth-restrictive conditions, they were washed once with serum-free RPMI-1640 medium, and then growth-arrest was induced in serum-free RPMI-1640 medium for 24 h to synchronize the cell growth. The podocytes were then ready for the following experiments.

### Cell stimulation

Differentiated mouse podocytes were stimulated with normal glucose [NG; 1 g/l D-glucose (Sigma, St. Louis, MO, USA)] or HG (4.5g/l D-glucose). A third group of podocytes was exposed to 1 g/l D-glucose plus 24.4 mM mannitol (Sigma) as an osmotic control (M). Cells in each group were collected at 12, 24, 48 and 72 h for analyses.

### TUNEL assay

Apoptotic cells were identified by the TUNEL technique (Promega, Madison, WI, USA), according to the manufacturer’s instructions. Differentiated mouse podocytes were plated on cover slides in six-well plates. Following stimulation with the indicated treatments, the cells were washed with 0.01 M phosphate-buffered saline (PBS) and fixed with 4% paraformaldehyde at room temperature for 30 min. The cells were then treated with proteinase K at room temperature and incubated in terminal deoxynucleotidyl transferase (TdT) buffer for 1 h at 37°C. Endogenous peroxidase activity was inhibited using 0.3% H_2_O_2_. The cells were incubated with horseradish peroxidase (HRP)-conjugated streptavidin at room temperature for 5 min. DAB working solution was added and the cells were counterstained with hematoxylin. Negative control cells were incubated with the labeling solution (without TdT) instead of the TdT reaction solution. Apoptotic cell nuclei appeared as dark brown/black under an E600 light microscope (Nikon, Tokyo, Japan). For the quantification of TUNEL-positive (apoptotic) cells, a minimum of 200 cells were counted at six random fields (×10) per group, and the percentage of the positively-labeled cells was calculated.

### Analysis of apoptosis by flow cytometry

The differentiated mouse podocytes were synchronized and stimulated with the indicated treatments for 12, 24, 48 and 72 h. The cells were collected and washed twice with 4°C normal saline. The supernatant was removed following centrifugation at 1,000 rpm for 5 min. The cells were then fixed with 70% ethanol overnight. The cells were centrifuged (1,000 rpm, 5 min), washed with normal saline, and then 1 ml DNA dye [propidium iodide (PI) 50 μg/ml, RNase 10 μg/ml and 1% Triton X-100; Sigma] was added. After staining for 30 min at 4°C, the cells were analyzed by flow cytometry (Epics-XL II; Beckman Coulter, Brea, CA, USA). Expo32ADC software was used for analysis: a hypo-diploid peak prior to the peak of the diploid cells was considered as the apoptotic cell population. The apoptotic rate was calculated according to the distribution histogram of the hypo-diploid population.

### Immunocytochemistry

PV-9000 two-step immunohistochemical reagent (Beijing Zhongshan Golden Bridge Biotechnology Co., Ltd., China) was used to assess GRP78, CHOP/GADD153 and caspase-12 expression. In brief, the podocytes were plated on cover slides in six-well plates. Following stimulation with the indicated treatments, the cells were fixed with 4% paraformaldehyde at room temperature for 15 min. Following pre-treatment with 0.3% Triton X-100 for 20 min at 37°C, the cells were blocked with goat serum for 30 min at 37°C. The cells were then incubated with rabbit anti-GRP78 polyclonal antibody (1:100 dilution; NeoMarkers Inc., Fremont, CA, USA), rabbit anti-CHOP/GADD153 polyclonal antibody (1:100 dilution; Santa Cruz Biotechnology Inc., Santa Cruz, CA, USA) and rabbit anti-caspase-12 polyclonal antibody (1:1,000 dilution; Abcam, Cambridge, MA, USA) overnight at 4°C. Following three washes with PBS, the cells were incubated with a polymer helper and polyperoxidase-anti-rabbit IgG (Beijing Zhongshan Golden Bridge Biotechnology Co., Ltd., Beijing, China) at 37°C for 30 min, and the cells were then stained with diaminobenzidine. A negative control was created by replacing the primary antibody with PBS buffer. The cells were counterstained with hematoxylin. The entire stained areas were scanned at ×100 magnification using an E600 light microscope.

### Reverse transcription polymerase chain reaction (RT-PCR)

Total RNA was extracted from each group using TRIzol reagent (Invitrogen). RNA purity was estimated by calculating the 260/280 nm absorbance and reverse transcribed into cDNA using an RT-PCR kit (Promega), according to the manufacturers’ instructions. Relative levels of target gene mRNA expression normalized to 18S rRNA were determined by the PCR system using the cDNA as a template and specific primers synthesized by Beijing AuGCT Biotechnology Co. Ltd., Beijing, China (Table I). PCR reactions were performed in duplicate at 95°C for 5 min and subjected to 36 cycles of 94°C for 1 min, 56–59°C for 45 sec and 72°C for 1 min, followed by 72°C for 10 min. PCR products were separated by 2% agarose gel electrophoresis with ethidium bromide staining and analyzed using a GDS-8000 Bioimaging system (UVP Inc., Upland, CA, USA) and LabWorks 4.5 software (UVP Inc.).

### Western blot analysis

The cells were harvested and homogenized on ice-cold homogenization buffer (10 mM Tris-HCl, pH 7.4, 1.5 mM EDTA, pH 8.0 and 100 mg/l phenylmethylsulfonyl fluoride; Sigma), followed by centrifugation at 20,000 × g for 20 min at 4°C. Supernatants were collected for characterizing the relative levels of protein expression by western blot analysis. In addition, nuclear proteins in some cells were extracted using nuclear and cytoplasmic protein extraction kits (KeyGen Biotech, Nanjing, China), according to the manufacturer’s instructions, followed by the quantification of protein concentrations using Coomassie brilliant blue.

Total cellular protein (100 μg/lane) or nuclear protein (60 μg/lane) were separated by 10% SDS-PAGE and transferred onto polyvinylidene fluoride (PVDF) membranes (Millipore Corp., Billerica, MA, USA). After being blocked with 5% non-fat dry milk in Tris-buffered saline/Tween-20 (TBST) buffer for 2 h, the membranes were incubated overnight at 4°C with rabbit polyclonal antibodies against GRP78 (1:1,000 dilution), caspase-12 (1:1,000) and β-actin (1:1,000; Biosynthesis Biotechnology Co., Ltd., Beijing, China), rabbit polyclonal antibodies against CHOP/GADD153 (1:500) and histone H1 (1:500; Boster Biotechnology, Wuhan, China). Subsequently, the membranes were incubated with horseradish peroxidase-conjugated goat anti-rabbit IgG (1:5,000; Amersham Biosciences, Piscataway, NJ, USA) at 37°C for 2 h. After TBST washing, enhanced chemiluminescence (ECL) reagent (Tiangen Biotech Co., Ltd., Beijing, China) was added to the membranes. Western blot bands were read for integrated optical density (IOD) and quantified using LabWorks 4.5 software (UVP).

### Statistical analysis

Data were processed using SPSS 13.0 software (SPSS Inc., Chicago, IL, USA). The results are presented as the means ± standard deviation (SD). Statistical analysis was performed using one-way ANOVA with the least significant difference (LSD) t-test for post hoc analysis. Correlation analysis was performed using the Spearman’s test. A value of P<0.05 was considered to indicate a statistically significant difference.

## Results

### HG induces apoptosis of differentiated mouse podocytes

**Figure 1:**
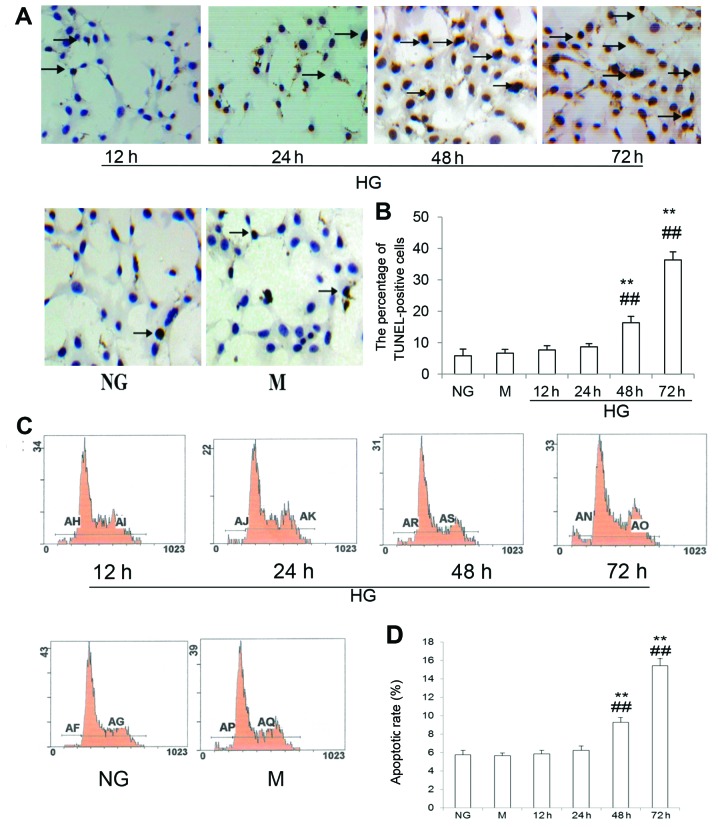
Effects of high glucose on differentiated mouse podocyte apoptosis. Differentiated mouse podocytes were incubated with 1 g/l D-glucose [normal glucose group (NG) group], 1 g/l D-glucose plus 24.4 mM mannitol (M group, osmotic control) and 4.5 g/l D-glucose [high glucose group (HG) group] for 12, 24, 48 and 72 h. (A) Apoptosis in podocytes was detected using the TUNEL assay. Arrows indicate TUNEL-positive (apoptotic) cells (magnification, ×100). (B) For quantification of TUNEL-positive (apoptotic) cells, a minimum of 200 cells was counted at six random fields (magnification, ×100) per group, and the percentage of the positively-labeled cells was calculated. (C) Apoptosis was determined by propidium iodide (PI) staining using flow cytometry. (D) The results of flow cytometry were expressed as the percentage (%) apoptotic rate. Apoptotic rate was calculated according to the distribution histogram of the hypo-diploid population. Data are presented as the means ± standard deviation (SD). ^**^P<0.01 vs. NG group; ^##^P<0.01 vs. M group.

To determine the effects of HG on podocyte apoptosis, the podocytes were cultured in RPMI-1640 medium containing 1 g/l D-glucose (NG, control group), 1 g/l D-glucose plus 24.4 mM mannitol (M group, osmotic control group) and 4.5 g/l D-glucose (HG, experimental group) for 12, 24, 48 and 72 h. Apoptotic rates were determined by TUNEL assay and flow cytometry (PI staining). Fig. 1A and B shows that compared with the control groups, podocyte apoptotic rates in the HG group were increased at 48 h, and even more so at 72 h. Indeed, 5.8±2.1% apoptotic cells in the NG group and 6.7±1.2% apoptotic cells in the M group, compared with 16.3±2.1 and 36.3±2.6% apoptotic cells in the HG group at 48 and 72 h (all P<0.01), respectively, were detected by TUNEL assay. The results were consistent with the results from flow cytometric analysis (Fig. 1C and D). As expected, mannitol had no effect on podocyte apoptosis as assessed using these two methods.

### HG stimulation upregulates the expression of the ER chaperone, GRP78, in differentiated mouse podocytes

**Figure 2:**
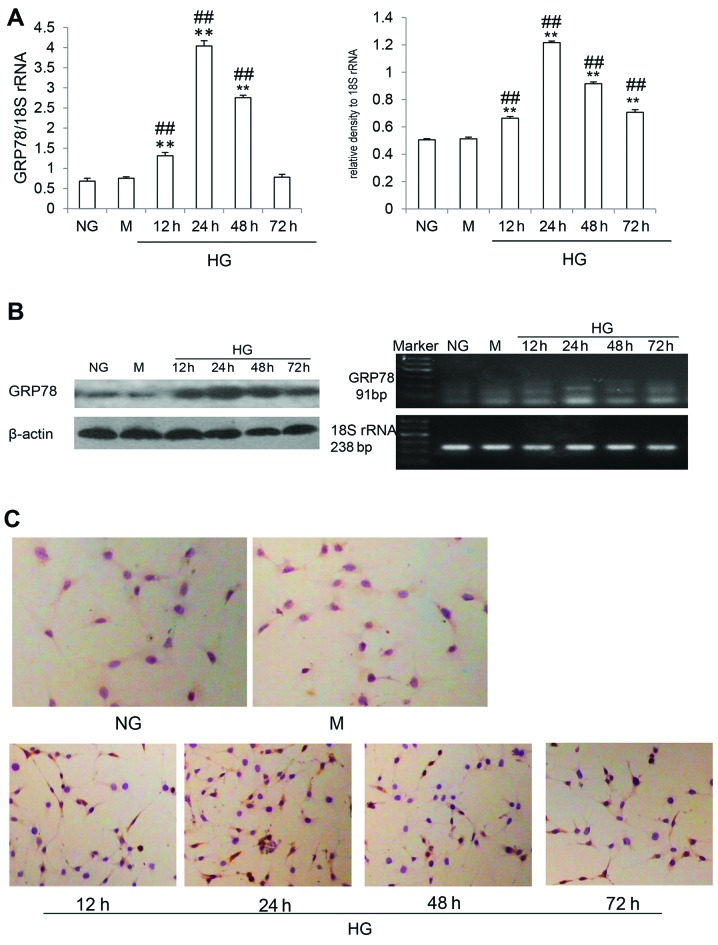
Effects of high glucose (HG) on GRP78 mRNA and protein expression on differentiated mouse podocytes. Podocytes were incubated with 1 g/l D-glucose [normal glucose group (NG) group], 1 g/l D-glucose plus 24.4 mM mannitol (M group, osmotic control) and 4.5 g/l D-glucose (HG group) for 12, 24, 48 and 72 h. (A) GRP78 mRNA expression was analyzed by RT-PCR, mRNA expression was normalized to 18S rRNA. (B) GRP78 protein expression was determined by western blot analysis. Protein expression was normalized to β-actin. (C) GRP78-positive expression was detected by immunocytochemistry (magnification, ×100). Data are presented as the means ± standard deviation (SD). ^**^P<0.01 vs. NG group; ^##^P<0.01 vs. M group.

GRP78, an important molecular chaperone localized in the ER, was used as an indicator of ERS (16). As can be seen in Fig. 2C, immunocytochemistry revealed that GRP78 was abundantly expressed in the cells in the HG group. By contrast, the cells in the NG and M groups showed only a modest or weak GRP78 signal. Western blot analysis revealed that GRP78 expression was significantly increased at 12 and 24 h (both P<0.05), and then decreased at 48 h, approximately returning to the control levels by 72 h (Fig. 2B). Furthermore, RT-PCR analysis revealed the same trend in GRP78 mRNA levels. However, GRP78 mRNA levels remained higher at 72 h (P<0.05) (Fig. 2A). These findings suggested that UPR was induced and that ERS was activated in the differentiated mouse podocytes exposed to HG.

### HG stimulation induces the activation of the CHOP/GADD153-dependent apoptotic pathway in differentiated mouse podocytes

**Figure 3:**
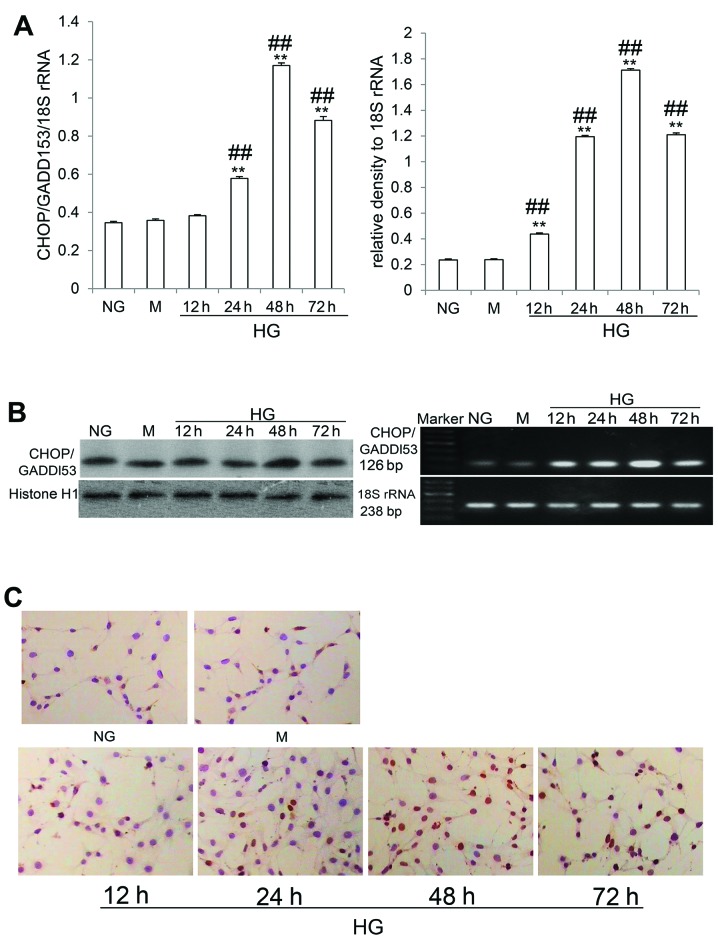
Effects of high glucose (HG) on CHOP/GADD153 mRNA and protein expression on differentiated mouse podocytes. Podocytes were incubated with 1 g/l D-glucose [normal glucose group (NG) group], 1 g/l D-glucose plus 24.4 mM mannitol (M group, osmotic control) and 4.5 g/l D-glucose (HG group) for 12, 24, 48 and 72 h. (A) CHOP/GADD153 mRNA expression was analyzed by RT-PCR, and the mRNA expression was normalized to 18S rRNA. (B) CHOP/GADD153 protein expression was determined by western blot analysis. Protein expression was normalized to β-actin. (C) CHOP/GADD153-positive expression was detected by immunocytochemistry (magnification, ×100). Data are presented as the means ± standard deviation (SD). ^**^P<0.01 vs. NG group; ^##^P<0.01 vs. M group.

CHOP/GADD153 is a nuclear protein forming stable heterodimers with C/EBP family members (8,17). As illustrated in Fig. 3C, it was detectable at low levels in the cells in the NG and M groups, whereas in the cells in the HG group, CHOP/GADD153 expression was increased as detected by immunocytochemistry. Western blot analysis revealed that CHOP/GADD153 expression was increased at 24 h, reaching a peak at 48 h, and then decreasing at 72 h, but without returning to the control levels (all P<0.05) (Fig. 3B). RT-PCR analysis revealed that CHOP/GADD153 mRNA levels were increased at 12 h, also reaching a peak at 48 h, and then decreasing at 72 h, but without returning to the control levels (all P<0.05) (Fig. 3A). These data suggested that the CHOP/GADD153-dependent apoptotic pathway was activated by HG stimulation in the differentiated mouse podocytes.

### HG stimulation activates the caspase-12-dependent apoptotic pathway in the differentiated mouse podocytes

**Figure 4:**
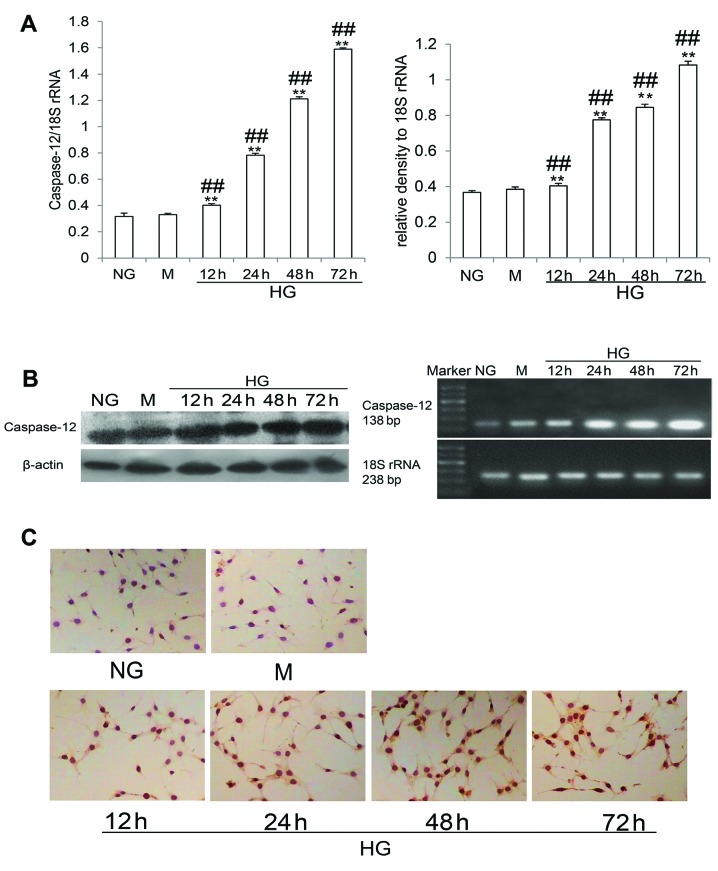
Effects of high glucose (HG) on caspase-12 mRNA and protein expression on differentiated mouse podocytes. Podocytes were incubated with 1 g/l D-glucose [normal glucose group (NG) group], 1 g/l D-glucose plus 24.4 mM mannitol (M group, osmotic control) and 4.5 g/l D-glucose (HG group) for 12, 24, 48 and 72 h. (A) Caspase-12 mRNA expression was analyzed by RT-PCR, and the mRNA expression was normalized to 18S rRNA. (B) Caspase-12 protein expression was determined by western blot analysis. Protein expression was normalized to β-actin. (C) Caspase-12-positive expression was detected by immunocytochemistry (magnification, ×100). Data are presented as the means ± standard deviation (SD). ^**^P<0.01 vs. NG group; ^##^P<0.01 vs. M group.

Caspase-12 is exclusively located in the ER. It is ubiquitously and constitutively expressed, but unlike other caspases, caspase-12 is specifically activated by ERS (18). As shown in Fig. 4C, immunocytochemistry revealed that caspase-12 expression was increased in the cells in the HG group compared with the control cells (NG and M group cells). Western blot analysis revealed that HG stimulation increased caspase-12 protein levels in a time-dependent manner (all P<0.05) (Fig. 4B). HG stimulation also increased the mRNA levels of caspase-12 in a time-dependent manner (all P<0.05) (Fig. 4A). These data indicated that HG stimulation activated the caspase-12-dependent apoptosis pathway in the differentiated mouse podocytes.

### Correlation between caspase-12 and CHOP/GADD153 protein expression and apoptosis induced by HG

Spearman’s correlation analysis revealed that caspase-12 and CHOP/GADD153 positively correlated with the apoptotic rate (r=0.915, P<0.01 and r=0.639, P<0.01).

## Discussion

Podocytes, a type of glomerular epithelial cell, are unique, highly specialized and terminally differentiated cells (6). The loss of podocytes leads to the stripping of areas of the glomerular basement membrane, which contributes to impaired renal function, as is evident by proteinuria and the development of glomerulosclerosis (7). It is generally accepted that apoptosis is a major cause of podocytes loss, occurring early in the development of DN and closely correlating with its progression (19,20).

ERS is generally present under physiological and pathological conditions, and is an important inducer of cell apoptosis (15,16). A growing number of studies have demonstrated that ERS plays a key role in the pathogenesis of several renal diseases, including DN (21). In the present study, we investigated the hypothesis that ERS is partly responsible for podocyte apoptosis induced by HG. We observed that some podocyte apoptosis was detected after 12 and 24 h in the cells exposed to HG and the control cells, but without significant differences between these two groups. However, the number of apoptotic cells markedly increased with the increasing exposure time to HG.

The levels of GRP78, a key UPR modulator (15), were increased in the cells exposed to HG at 12 and 24 h, and decreased by 72 h. These results suggested that UPR was induced and that ERS was activated when the podocytes were exposed to HG. Based on current knowledge, the function of UPR is to adapt to the changing environment and to re-establish a normal ER function (15). However, our data indicated that this protective effect of the UPR lasted only for a short time, even if the stress was persistent. These results suggest that this protective mechanism is eventually overpowered and that apoptosis ensues.

CHOP/GADD153 is a nuclear protein that forms stable heterodimers with C/EBP family members (22). It is barely detectable under normal physiological conditions, but it is strongly induced in response to ERS (23). The overexpression of CHOP/GADD153 and the microinjection of CHOP/GADD153 protein have been reported to lead to cell cycle arrest and/or apoptosis (24–27). The overexpression of GRP78 (also known as BiP) may attenuate the induction of CHOP/GADD153 in ERS and may reduce ERS-induced apoptosis. Our results revealed that just as GRP78 expression began to weaken at 48 h, CHOP/GADD153 expression reached its peak. Furthermore, CHOP/GADD153 expression correlated with the apoptotic rate in cells exposed to HG.

Caspase-12 is exclusively located in the ER (18). It is ubiquitously and constitutively expressed, but unlike other caspases, caspase-12 is specifically activated by insults inducing ERS and not by other death stimuli (29). Following its activation, it can directly process downstream caspases in the cytosol, mainly caspase-9 and -3 (30). Our results demonstrated that caspase-12 expression gradually increased with time in response to HG, and that its levels correlated with the apoptotic rate in cells exposed to HG.

The results from the present study demonstrated that hyperglycemia induced ERS in podocytes, and that this stress gradually exceeded the capacity of different protective mechanisms, including GRP78 response. CHOP/GADD153 and caspase-12 were activated according to different patterns, suggesting that these mechanisms contribute differently to podocyte apoptosis. Nevertheless, these mechanisms partially contribute to the pathogenesis of DN.

In conclusion, hyperglycemia induced apoptosis partly through ERS in differentiated mouse podocytes, and this may contribute to the pathogenesis of DN. HG-stimulated podocytes undergo ERS, which presumably is an adaptive, protective UPR reaction for cell survival; however, this protective effect was short-lived, since continued exposure to HG eventually overpowered this effect and led to apoptosis. These results indicate that novel (previously unknown) mechanisms involved in DN may be targeted by novel therapeutic interventions.

## Figures and Tables

**Table I:** PCR primer sequences.

Gene	Primer sequences	T_m_ (°C)	Product size (bp)
GRP78	F: 5′-AACCCAGATGAGGCTGTAGCA-3′ R: 5′-ACATCAAGCAGAACCAGGTCAC-3′	55	91
CHOP/GADDl53	F: 5′-CCAGCAGAGGTCACAAGCAC-3′ R: 5′-CGCACTGACCACTCTGTTTC-3′	42	126
Caspase-12	F: 5′-CACTGCTGATACAGATGAGG-3′ R: 5′-CCACTCTTGCCTACCTTCC-3′	56	138
18S rRNA	F: 5′-ACACGGACAGGATTGACAGA-3′ R: 5′-GGACATCTAAGGGCATCACA-3′	56	238

F, forward primer; R, reverse primer; T_m_, melting temperature; PCR, polymerase chain reaction; GRP78, glucose-regulated protein 78; CHOP/GADDl53, CCAAT/enhancer-binding protein (C/EBP) homologous protein (also known as GADD153).
